# Supplementary material for: CD200R1 promotes IL-17 production by ILC3s, by enhancing STAT3 activation
Source: Mucosal Immunol. Author manuscript; Available in PMC 2023 Jul 26. (PMC10270648; doi:10.1016/j.mucimm.2023.01.001)
Supplement: Supplementary Information [file EMS181071-supplement-Supplementary_Information.pdf]

Supplementary Information

Supplementary methods

Skin infection model

C. albicans SC5314 (provided by Dr Andrew Sharp) was grown on Sabouraud agar (SAB) plates supplemented with 50 μg/mL chloramphenicol (Sigma Aldrich, Gillingham, UK) overnight. Colonies were cultured for 18-24 hours at 37°C in yeast extract-peptone-dextrose (YPD) broth (MP Biomedicals, Santa Ana, CA, USA). The fungi were washed with PBS and 2 x 10^7 CFU was applied topically to shaved dorsal skin. After 48hr mice were euthanised and analysed. Fungal burden was determined by lysing small skin pieces in Precellys hard tissue lysing tubes (Stretton Scientific, Alfreton, UK) containing PBS and 1% penicillin and streptomycin, using a Precellys 24 homogenising instrument (Stretton Scientific, Alfreton, UK). Lysate was cultured on YPD plates supplemented with 50 μg/mL chloramphenicol at 30°C for 24 hours before colony enumeration.

Supplementary Table 1

Antibodies used for flow cytometry

Antibody specificity	Clone	Manufacturer
B220	RA3/6B2	eBioscience, Waltham, MA, USA
CCR2	475301	R & D systems, Minneapolis, MN, USA
CCR6	29-2L17	Biolegend, San Diego, CA, USA
CD1d	1B1	Biolegend
CD3ε	145-2C11	eBioscience
CD4	RM4-5	Biolegend
CD11b	M1/70	eBioscience
CD11c	N418	eBioscience
CD19	eBio1D3	eBioscience
CD24	M1/69	Biolegend
CD25	PC61	Biolegend
CD45	30-F11	eBioscience
CD45.1	NDS58	BD Bioscience, Franklin Lakes, NJ, USA
CD45.2	104	eBioscience
CD64	X54-5/7.1	Biolegend
CD90.2	53-2.1	eBioscience
CD117 (cKit)	2B8	eBioscience
CD127	A7R34	eBioscience
CD200R1	OX110	eBioscience
CD207	eBioL31	eBioscience
F4/80	BM8	eBioscience
FcεRIα	MAR-1	eBioscience
GATA3	TWAJ	eBioscience
Gr1 (Ly-6G/Ly-6C)	RB6-8C5	eBioscience
IL-17A	eBio17B7	eBioscience
IL-23R	3C9	BD Biosciences
Ly6C	HK1.4	eBioscience
Ly-6G	1A8	BD Biosciences
MHCII (IA-IE)	M5/114.15.2	eBioscience
NKp46	29A1.4	eBioscience
RORγt	B2d	eBioscience
ScaI (Ly6A/E)	D7	Biolegend
TCRβ	H57-597	BD Bioscience
TCRγδ	eBioGL3	eBioscience
Ter119	TER-119	eBioscience

Figure S1

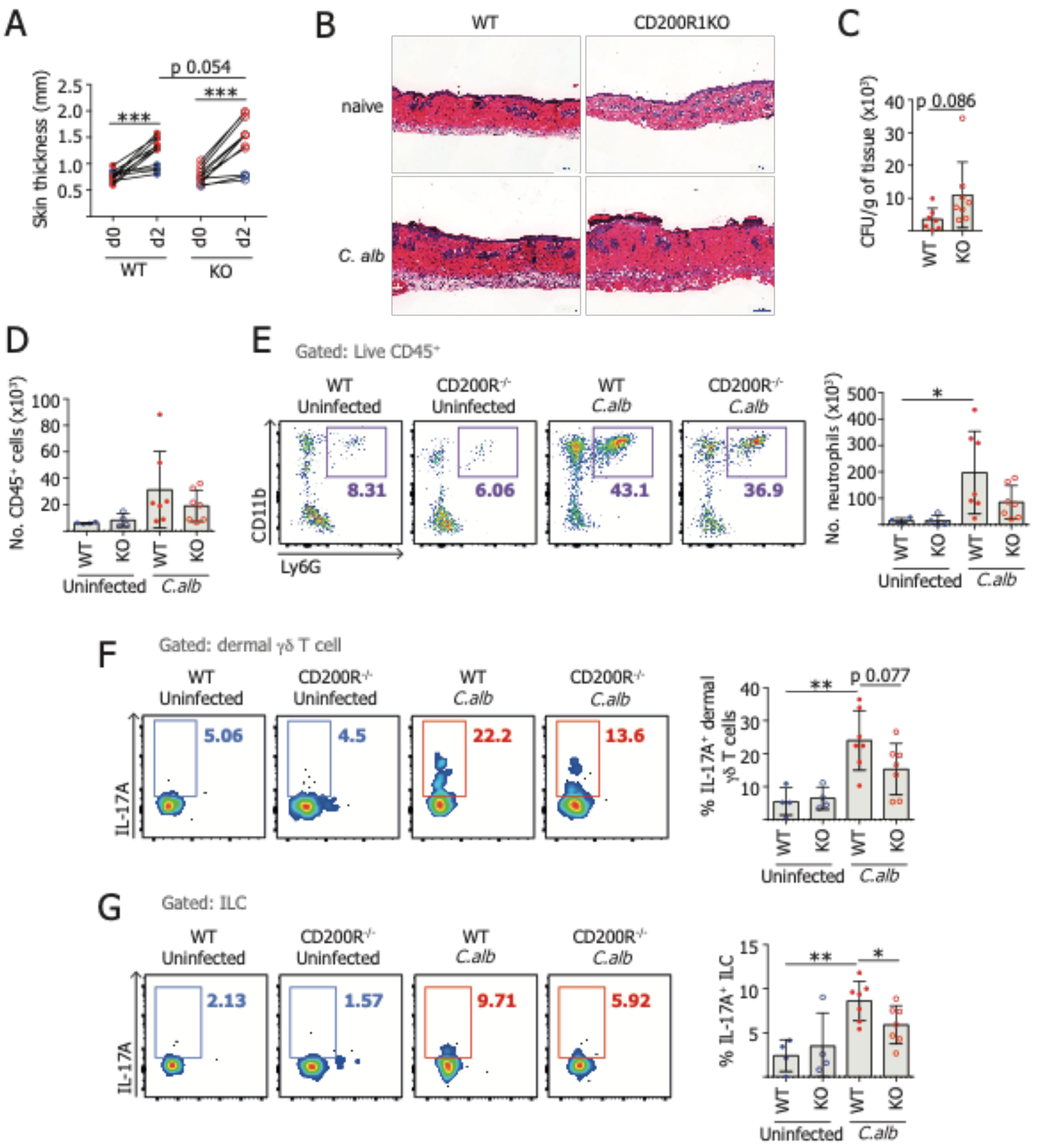

Figure S1: CD200R1 deficiency reduces the IL-17A production by ILCs in response to *C. albicans* skin infection

WT and CD200R1KO (KO) mouse dorsal skin was infected with 10^7 CFU *C. albicans SC5314* (or PBS) and after 2 days mice were euthanised and disease severity was analysed. **A.** Skin thickening measured using a digital micrometer. **B.** H&E stained skin sections. **C.** Fungal burden in skin. **D.** Number of live single cells that are $CD45^+$ in infected and uninfected skin. **E.** Number of $CD45^+$ cells that are neutrophils in infected skin. **F.** Proportion of skin $CD3^{low}$ $\gamma\delta$ T cells producing IL-17A. **G.** Proportion of skin ILCs producing IL-17A. Data shown are from 2 pooled independent experiments. A, D-G Data were analysed by two-way ANOVA followed by a Bonferroni post hoc test. C data were analysed by Student's t test.

Figure S2

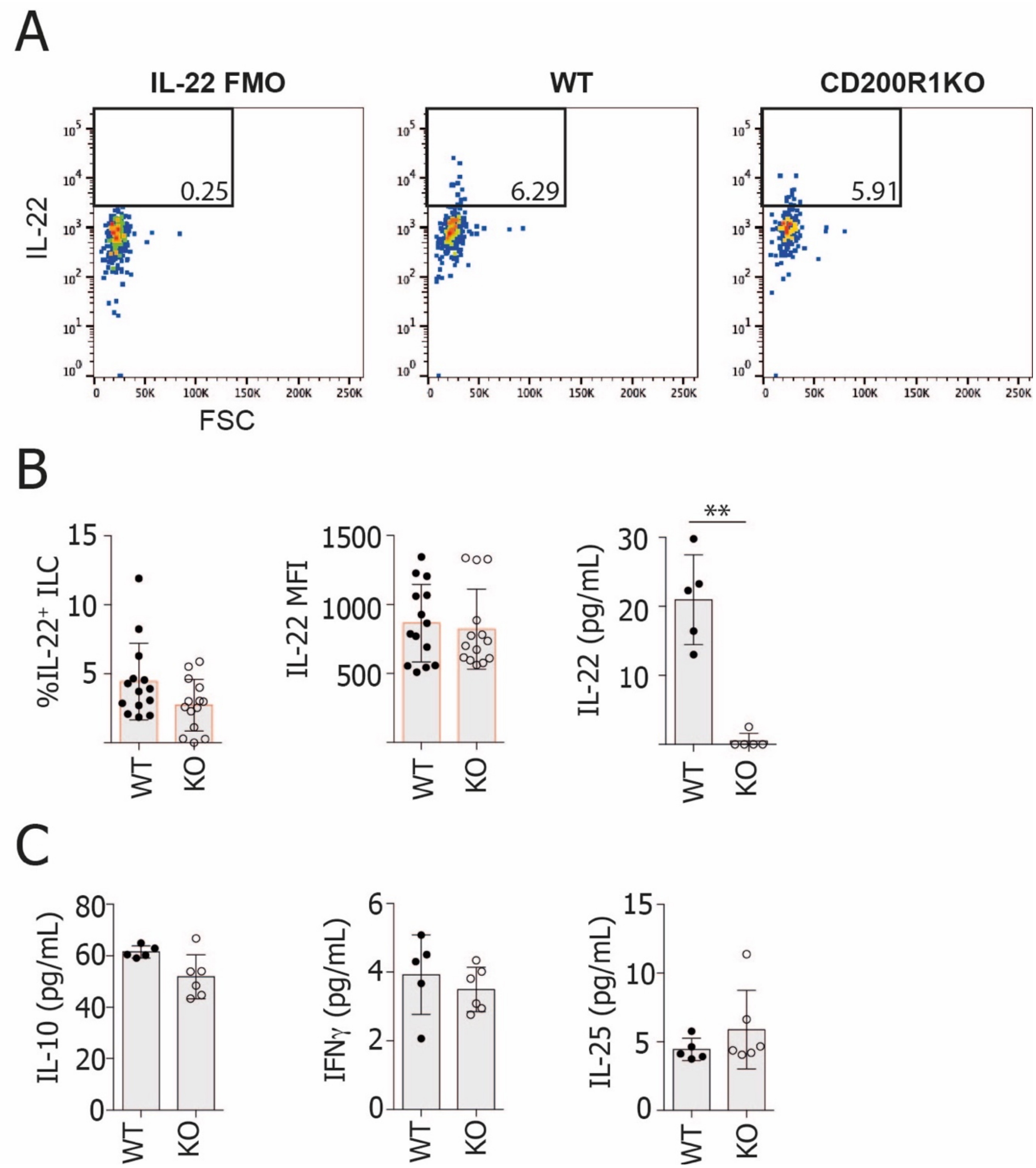

Figure S2: The effect of CD200R1 on IL-22 production by ILC3 is unclear, but known ILC3-inhibitory cytokines are not elevated in CD200R1KO skin cell cultures

Total dorsal skin cells were isolated from WT and CD200R1KO (KO) mouse dorsal skin and were stimulated with IL-23 overnight. **A-B**. IL-22 production was analysed in ILCs by flow cytometry, and in the culture supernatant by ELISA. **C.** IL-10, IFNγ and IL-25 levels were measured in culture supernatant by ELISA. Data are from two (B) or one (C) experiment, representative of two independent experiments. Data were analysed by Student's t test.

Figure S3

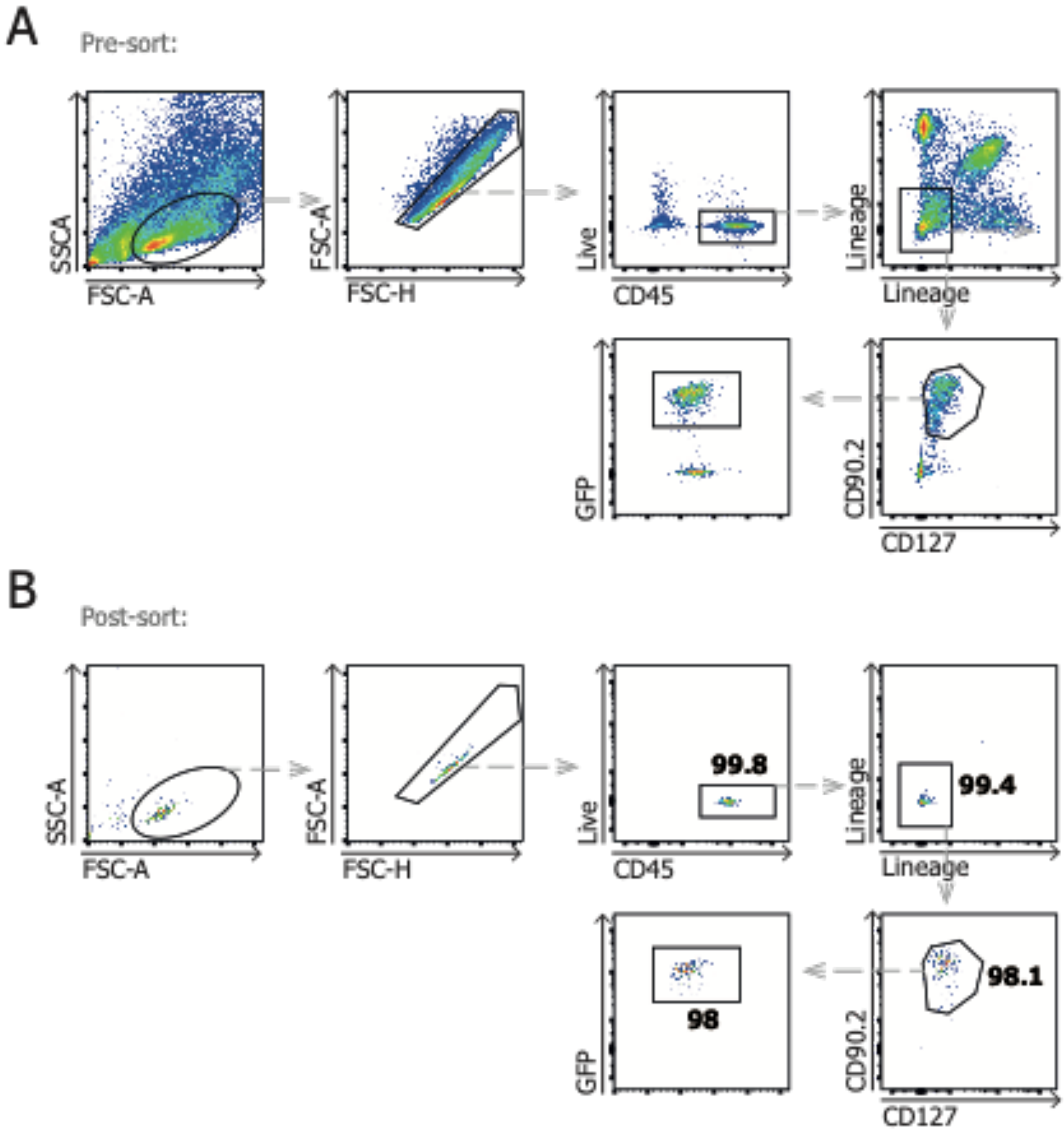

Figure S3: Flow cytometric sorting of Rorc eGFP+ ILC3s

Small intestinal lamina propria cells were isolated, stained with antibodies and were sorted using an Aria flow cytometer. **A.** Cells prior to flow cytometric cell sorting and **B.** Purity of the sorted cells ($CD45^+$ $Lineage^-$ $Thy1^+$ $CD127^+$ RORc $eGFP^+$). Data shows representative flow cytometric sorting gating and purity.
